# Toe-divided Shoes for Contracted Hoofs

**Published:** 1898-06

**Authors:** M. J. Treacy

**Affiliations:** Veterinarian, United States Army, Fort Meade, South Dakota


### TOE-DIVIDED SHOES FOR CONTRACTED HOOFS.

By M. J. Treacy, M.R.C.V.S.,

VETERINARIAN, UNITED STATES ARMY, FORT MEADE, SOUTH DAKOTA.

An article appeared in a recent English journal advocating the above. Their advantages are apparent to the tyro. Their retention on the feet was a very doubtful factor to the writer. The following experiments were instituted: These shoes were fitted as usual, then heated at the toes and divided by a cold chisel; the consequent roughness filed smooth ; they were then applied slightly apart at the toes, say one-eighth of an inch. One additional toe-nail was used in each half where heel or toe-calking was applied. These shoes must be fitted hot.

On the third day of January there were shod after the above fashion the following animals affected with contracted hoofs : One driving team doing ten miles daily, heel-calking; one driving team doing twenty miles daily, flat shoes; two saddle horses doing ten miles daily, heel- and toe-calking; one draught-mule doing fifteen miles daily, heel- and toe-calking. On February 17th, or after six weeks' daily work, these shoes were removed, and were quite firmly attached to the feet. The work was performed on country roads and at all gaits.

All the feet had spread at the heels from one-half inch to one inch. All the front toes of the shoes had been forced together by the heel expansion, where they had been originally left one-eighth of an inch apart. The frogs were enlarged and expanded. The animals were all improved visibly in their gait, and the majority of them were sound. I have some curiosity as to how these shoes would be retained on pavements, and would like to hear from some city confrères.

My private team was shod with toe-divided shoes, and I will use no others, unless I am compelled to do so by unforeseen circumstances, as one of them, a valuable standard-bred driver and family friend, has become sound by the use of these shoes, although I bought him as a " sore horse " five years ago. After two shoeings with the above he is now sound, his heels having spread over one and a half inches at time of writing. Three out of four cases of obscure lameness responded favorably to the application of toe-divided shoes and retention at labor. And I have never failed so far to see a contracted foot which did not expand visibly, more particularly when the shoes were flat (without toe-pieces and calking), the feet soaked and pressed daily and frog and bar ground-contact obtained. Even in well-marked cases of navicular disease, where frog-pressure cannot be tolerated, there is a marked improvement in the movements after their application.

---

The veterinarians of Austria are preparing to make a collection of their writings that have appeared during the reign of the present Emperor.

